# Meeting the Challenge of Diabetes in Ageing and Diverse Populations: A Review of the Literature from the UK

**DOI:** 10.1155/2016/8030627

**Published:** 2016-10-17

**Authors:** Emma Wilkinson, Muhammad Waqar, Alan Sinclair, Gurch Randhawa

**Affiliations:** ^1^Institute for Health Research, University of Bedfordshire, Luton, UK; ^2^Foundation for Diabetes Research in Older People, Diabetes Frail, Droitwich, UK

## Abstract

The impact of type 2 diabetes on ageing societies is great and populations across the globe are becoming more diverse. Complications of diabetes unequally affect particular groups in the UK older people, and people with a South Asian background are two population groups with increased risk whose numbers will grow in the future. We explored the evidence about diabetes care for older people with South Asian ethnicity to understand the contexts and mechanisms behind interventions to reduce inequalities. We used a realist approach to review the literature, mapped the main areas where relevant evidence exists, and explored the concepts and mechanisms which underpinned interventions. From this we constructed a theoretical framework for a programme of research and put forward suggestions for what our analysis might mean to providers, researchers, and policy makers. Broad themes of cultural competency; comorbidities and stratification; and access emerged as mid-level mechanisms which have individualised, culturally intelligent, and ethical care at their heart and through which inequalities can be addressed. These provide a theoretical framework for future research to advance knowledge about concordance; culturally meaningful measures of depression and cognitive impairment; and care planning in different contexts which support effective diabetes care for aging and diverse populations.

## 1. Introduction 

**Figure 1:**
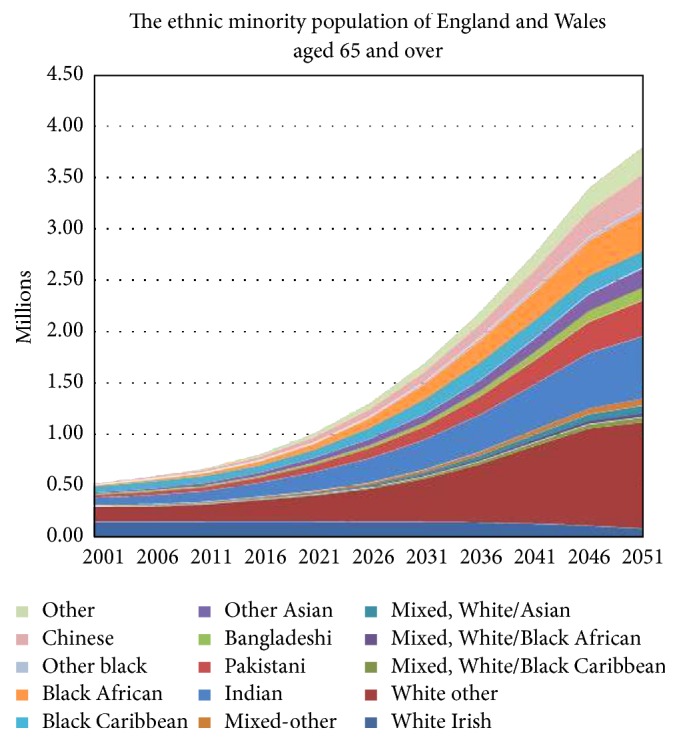
Ethnic minority population projections to 2051, England and Wales from Lievesly, 2010 [4].

Although longevity in the UK is increasing, average increases mask important differences within the population [1–3]. Furthermore as the UK population as a whole grows older, the demographics within it are changing (see Figure 1). Currently most ethnic minorities have younger populations than the majority White British population. However by 2051, the ethnic groups with the highest proportions of people, aged 50 and over will be “Other White,” Chinese, “Other Asian,” Indian, “Other,” and White Irish alongside White British. In the “non-White” ethnic group alone, there will be 2.7 million people aged 65 and over and 1.9 million people aged 70 and over [4]. Whilst ethnic minorities already make up around half the local population in some parts of the country, by 2056 they will make up 43 percent of the total national population [5]. Together these changes highlight the need to focus attention to commissioning health services for an increasingly multiethnic older population.

The purpose of this research was to review the evidence to guide a programme of applied research to address the key areas and processes for reducing inequalities in diabetes care for older people from ethnic minority groups. We focussed in this instance on South Asians in the UK as this group has an established history in the UK (making up fifty percent or more of the population in some UK locations) and so would be likely to feature in the relevant research literature.

We define ethnicity as a consciousness of belonging to a particular group based on commonality of family origin and culture of shared values and beliefs which is socially constructed [6] and loosely related to country of birth, ancestral country of birth, language spoken at home, nature of geographical origin, racial group, and religion [4]. The broad South Asian ethnic group descriptor used in this review (unless otherwise stated) refers to the majority South Asian populations in the UK: Indian Punjabi, Indian Gujarati, Bengali, Pakistani, and Sri Lankan.

The term “older people” is used variously according to context and different age-related dimensions: chronological, biological, functional, psychological, and social. In western societies, it broadly aligns with age of retirement 60 or 65+ [7, 8] but this is a socially constructed time point which does not take into account other factors relevant to diabetes care and ethnicity such as the onset of complications; and we account for this in our strategy for searching the literature.

This study's principal interest was diabetes care and prevention in relation to inequality, ethnicity, and the older population rather than the aetiology of diabetes per se, although this was necessarily touched on where it related to clinical practice and management of older people who are living with diabetes. Diabetes care in the UK is a context specific and complex activity because it takes place across NHS settings through consultations in primary and secondary care and in people's home through self-management and care support.

Inequality was the main lens through which the literature review was conducted. The starting point was that our previous research in related areas had found inadequate care of older people with diabetes particularly those being cared for in residential settings [9]; that providing equitable care through the diabetes care pathway was a challenge for care providers [10]; and that people with a South Asian background and diabetes can be doubly disadvantaged by having increased risk of developing diabetes compared to people with a White European background in the UK and additional access barriers [11]. These separate but related findings suggested to us that it was important to look at the evidence as a whole and to understand the processes which could help inform action on inequalities.

Although familiar with some of the concepts associated with reducing inequalities in access to diabetes care, such as cultural competency and concordance [12], we had not considered these specifically in relation to older people and the increasingly diverse and ageing UK population before. This was the first review, as far as we were aware, to do so and it was by nature and design exploratory. We used a realist review methodology to help us search the literature and to start to build a theoretical base for our research programme.

The review had two parts: Part 1, a mapping phase where we thematically synthesised the relevant studies into the main areas of research evidence; and Part 2, a theory building phase where we hypothesised, by abstracting from the evidence, a theoretical framework for moving forward from this base. As the work was early stage the emergent theory raised further questions which will help test and refine the theory in the future. As it stands however the review highlights a number of issues for policy makers, providers, and researchers concerned with reducing and preventing inequalities in diabetes care and these are summarised at the end of this paper.

## 2. Methodology and Methods

**Figure 2:**
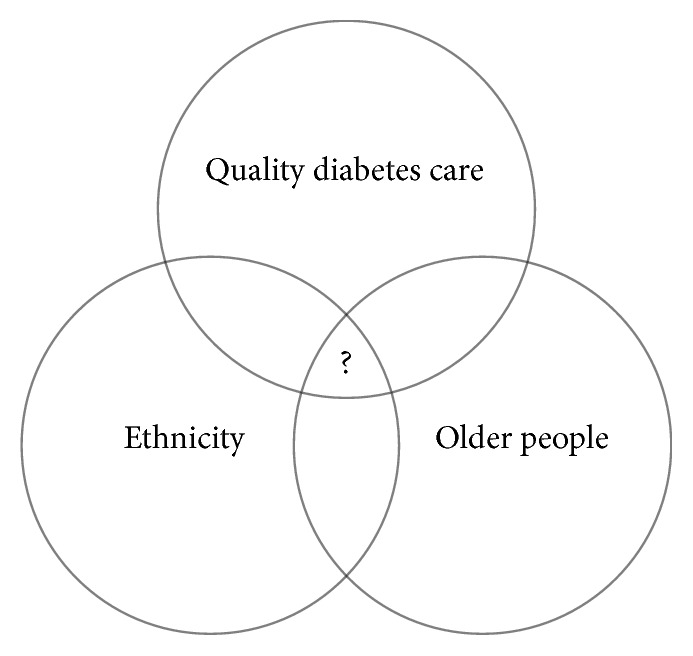
Areas for literature review.

We reviewed literature at the intersection of three areas: quality diabetes care, older people, and ethnicity (see Figure 2). The review was conducted by a multidisciplinary team comprising researchers with interest and expertise in public health, diabetes, gerontology, and diversity research.

Following an initial exploratory phase we decided to apply a realist approach to review the literature. This methodology was considered the most appropriate because it accommodated the broad research question; was compatible with the complex and context related nature of diabetes care; was sympathetic to the usage of a multimethod, multidisciplinary evidence base; and would facilitate the exposition of theory through emerging and generalisable mechanisms [13]. This could inform our programme of research but also be useful to policy makers and practitioners working with other ethnic minority groups in the UK.

### 2.1. Literature Search

We conducted an initial scoping phase in which we hand-searched for research publications and tested different search strategies with available electronic databases. We made a number of decisions about the search strategy which are listed as follows:

1. The search would be limited to the previous 30-year timeframe and to studies from the UK. The period 1985 to 2015 spanned several changes of UK governing parties and associated health policies some of which addressed health inequalities in relation to diabetes care, the impact of which would be captured in literature published during this time.
2. The search would focus specifically on the UK population. Ethnic minorities and health systems are different in different countries and diabetes care is context related. Literature on ethnicity, access, and cultural competency from other countries such as the US, Canada, and Australia was drawn on where appropriate in the analysis and discussion of mechanisms.
3. The search would focus on the UKs' South Asian population rather than other or all ethnic minorities living in the UK. Previous research by members of the team provided insight into some of the inequalities that people with a South Asian background experience [14]. As these related to this particular population group's migration and settlement in the UK they are likely be reflected in UK evidence from this timeframe. Despite a focus on one group we anticipated that some of the concepts and mechanisms emerging from this review would be applicable to other minority communities.
4. The search would use common age descriptors for older people as well as specific age categories from 55 years upwards. In the context of diabetes and care for minority groups age as a descriptor could be relative and variable depending on the population and phase of care (i.e., prevention, treatment, and palliation).
5. The search would use descriptors for diabetes that included the key complications: diabetic neuropathy, retinopathy, and nephropathy. Terms for the latter would be expanded as studies of diabetic nephropathy and end stage kidney disease would be likely to include the older South Asian population because of the links between ethnicity, diabetes, chronic kidney disease, and longevity [11, 15].
6. The search would be inclusive of research using qualitative and quantitative methods as well as grey literature in line with the realist methodology to prioritise relevance and contribution to theory building [16]. The quality standards applied in assessing potential publications were based on those appropriate for the type of publication, intervention, method, and design described [16, 17].

### 2.2. Searching Methods

A combination of hand searching and electronic searching of publication databases was carried out.

The following databases were searched: Academic Search Elite, CINAHL Plus with Full Text, MEDLINE, MEDLINE with Full Text, PsycARTICLES, PsycINFO, SocINDEX with Full Text, and Global Health. Publication abstracts were searched using keyword criteria as follows:

 Diabetes OR diabetes mellitus OR type 1 diabetes OR T1DM OR type 2 diabetes OR T2DM OR hyperglycem^*∗*^ OR hypoglycem^*∗*^ OR non insulin dependent diabetes mellitus OR NIDDM OR insulin OR insulin resistance OR glucose level OR glucose regulation OR haemoglobin A1c OR HbA1C OR metabol^*∗*^ OR foot problems OR amputation OR lower extremity OR lower limb OR complications OR nephropathy OR retinopathy OR kidney disease OR chronic kidney disease OR renal OR renal impairment OR kidney damage OR albuminuria OR proteinuria OR microalbuminura OR renal replacement therapy OR CKD OR ESRD OR ESRF OR ESKD OR ESKF OR RRT OR end stage renal disease OR end stage kidney disease OR end stage kidney failure OR end stage renal failure OR dialysis OR primary care AND Older people OR older persons OR elder^*∗*^ OR old age OR ageing OR aging OR late life OR frail^*∗*^ OR non frail OR end of life OR geriatrics OR gerontology OR post menopausal OR over 55 years OR over 60 years OR over 65 years OR over 70 years OR over 75 years OR over 80 years OR over 85 years OR over 90 years OR over 95 years OR over 100 years OR end of life OR functional disability OR functional decline OR mortality AND Ethn^*∗*^ OR race OR culture OR BAME OR BME OR minorit^*∗*^ OR ethnic minority OR asia^*∗*^ OR Indoasia^*∗*^ OR south asia^*∗*^ OR Indian OR Pakistani OR Bangladeshi OR Sri Lankan OR racial OR black^*∗*^ OR culturally and linguistically diverse group OR CALD

Electronic search results were screened for duplication and relevance to the review area and question. Copies of the full publication were obtained for included abstracts which were screened and those considered relevant included in the analysis. This process was conducted by EW and MW jointly, with assistance from an information specialist and with input and oversight from the other members of the author team.

### 2.3. Analysis and Synthesis

The research question “What are the key mechanisms for reducing inequalities in diabetes care in the UK for older people with a South Asian background?” was the basis of capturing learning from the published literature. In realist terms it was conceptualised as a complex intervention comprising government policy, applied research, and evidence based practice from the UK which addressed inequalities in diabetes outcomes and care for older people with diabetes from ethnic minorities and spanned the diabetes care system as a whole. We drew on the RAMESES guidance [18] for reporting realist review to help make our review and its findings as clear as possible.

The realist programme theory developed iteratively through the scoping, mapping, and theory building stages of the literature review and the findings are reported in two parts in Section 3:

 Part 1: the overview and mapping of literature relevant to the research question and search criteria.
 Included studies were analysed thematically into broad areas.
 Part 2: the building of a theoretical framework for research in response to the question.
 For each mapped area we considered the context, mechanisms, and outcomes and considered how these related to common concepts also emerging from the literature that could be explanatory in terms of observed inequalities in diabetes care and interventions to reduce them (see Table 1).

## 3. Results

**Figure 3:**
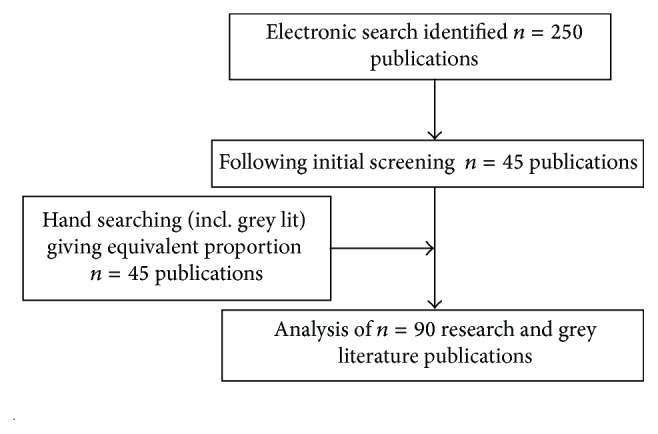
Search results.

The results of the literature search are summarised in Figure 3. The electronic search was most recently conducted on 27th July 2015.

### 3.1. Results: Part 1 Mapping

Following thematic analysis the included literature was following broad areas.

#### 3.1.1. Age and South Asian Ethnicity in Diabetes Research and Policy: Demography and Inequality

There were very few studies which specifically investigated diabetes, older people, and ethnicity, and even fewer (none) which specifically addressed diabetes care for older South Asian people in the UK. Although studies which included South Asian people with diabetes often stated in their background that diabetes was a leading cause of mortality and morbidity and South Asians were the largest ethnic minority in the UK, the majority of studies identified by our literature search concerned prevalence and incidence of diabetes, diabetes related complications, and associated conditions particularly cardiovascular disease. Exceptions to this were the UK Asian Diabetes Study (UKADS) [19] and the Prevention of Diabetes and Obesity in South Asians (PODOSA) [20] which were intervention studies of enhanced diabetes care and prevention respectively, within the UK South Asian population. We found however that the data and findings concerning age within included studies tended to be embedded within the results section of the publication, not detailed in the aims of the research nor discussed in more than a cursory way in relation to the timing of interventions in the population being studied.

In South Asians, the prevalence of type 2 diabetes is 4 times greater than that of White Europeans [21]. Most of the research papers which focussed on ethnicity included it as a demographic descriptor and independent variable of the outcome or outcomes being examined. Policy documents for diabetes, kidney care, and care of older people in contrast highlighted ethnicity as a key variable associated with inequality in access to quality care and in terms of interventions being culturally acceptable [22–24]. Recent guidelines for diabetes care for older people [25, 26] suggested that care should be individualised within an overarching theme of person centred diabetes care and that it should be tailored to individuals taking into consideration relevant factors. One such factor could be the person's ethnicity, but this was not explicitly stated within the guidelines.

Where ethnicity was discussed in relation to inequalities in the research literature it was mainly to explain variations in outcomes or patterns of distribution within a given population and there was a dearth of studies which analysed inequalities as it related to diabetes care specifically for older people with a South Asian ethnic background. There were very few studies about diabetes and diabetes care which explicitly included older South Asian people as participants and a similar number of papers which discussed the lack of participation of older people and ethnic minorities in studies as a research issue [27, 28].

#### 3.1.2. South Asians and Earlier Onset of Diabetes and Complications

Studies of diabetes which include an analysis by ethnicity invariably noted the earlier onset of diabetes in South Asians compared to White Europeans as an important factor in understanding both aetiology and disease progression as well as indicating a timeframe for intervention and prevention which is different to the majority population. South Asians experience diabetes approximately 10 years before White Europeans and show signs of more rapid progression of complications [29, 30]. Research studies of diabetes complications in ethnicity minorities did not explicitly identify older people for inclusion, but because complications are related to time since diabetes diagnosis and age, they included a large proportion of older people within their study populations “by default” [31, 32].

Together, key UK government guidelines, the Quality Outcomes Framework and the National Service Frameworks for Diabetes and Kidney Disease, have encouraged GPs to consider ethnicity as a factor for earlier diagnosis and targeted care. These quality initiatives have gone some way to redress inequalities in diabetes care [33] but there are concerns that, as they stand, they may perpetuate the existing status quo and not reduce inequalities further [34].

#### 3.1.3. South Asian Ethnicity, Heterogeneity, and Cardiovascular Disease

Several of the studies which detail South Asian ethnicity describe the heterogeneity within the broad South Asian descriptor for the UK's diverse South Asian population and some, depending on the data source, were able to break down their results across the main South Asian groups (Indian, Pakistani, and Bangladeshi) in the UK [35]. Ethnicity was linked to socioeconomic status in some studies including use of income level as a proxy for age as an alternative explanatory variable to capture some of the social and cultural associations with age.

The complicating associations between diabetes and cardiovascular disease (CVD) were the subject of over half the studies identified through our electronic search. These were seeking to understand the aetiology of morbidity and mortality of CVD and included diabetes and ethnicity as established risk factors in the analysis [36]. Similarly, in relation to high blood pressure and atherogenic lipid profile, key risk factors for circulatory diseases, these have been found to have an association with South Asian ethnicity both in comparison with other ethnic groups and amongst the main UK South Asian groups [37].

Differences in diabetes related mortality and morbidity between different ethnic groups outlined in a small number of publications point to different mechanisms through which ethnicity exerts influence. For example, South Asian and Black groups both have increased risk of diabetes, CVD, and Stroke [38] compared to White Europeans but show differences in level of risk and type of stroke. This in turn suggests particular genetic differences in addition to social and behavioural factors all or some of which may be linked [39]. Furthermore these studies have shown that when diabetes and age are controlled for, ethnicity exerts an independent effect on cardiovascular outcomes [40, 41].

#### 3.1.4. Diabetes and Complications Affecting Older South Asian People

Studies which focus on diabetic nephropathy show that South Asians also experience complications at an earlier age and their progression is faster than in White Europeans. South Asians' risk of diabetic nephropathy is 13 times that of the White European population [21]. As a group they are disproportionately represented in the population for renal replacement therapy, and because of this and the additional and independent risk of mortality from CVD that chronic kidney disease confers, together with a lack of ethnically compatible kidneys for transplantation, they are disproportionately represented in the group of people in need of end of life care [42].

Other diabetes complications, retinopathy and neuropathy, have a similar association with ethnicity; that is, they have been found to be associated with increased risk factors [43] and are indicators of microvascular damage. Furthermore South Asian populations are at increased risk of developing vascular dementia because of the increased incidence of diabetes, hypertension, and chronic kidney disease [44–46]. There is a higher rate of cognitive impairment in older people with CKD; it is largely unidentified and associated with severity of CKD [47–49].

As the South Asian population is ageing and as longevity is main risk factor for comorbidities in older people, the incidence of end stage renal failure and dementia are set to increase in South Asian ethnic group [50]. Both these complications are ultimately life limiting but have a disease trajectory which can last many years, and as retinopathy and neuropathy affect sight and pain symptoms, care provision of older people with diabetes and complications incorporates preventive activity, treatment of symptoms, and comorbidities and end of life care [22–24], which in the case of diabetic nephropathy may include renal replacement therapy.

Depression as a comorbid condition for people with diabetes is associated with both increased risk of developing cardiovascular problems as well as being secondary to cardiovascular complications and increasing risk of mortality [51]. It is also a prevalent and costly burden to end stage renal patients [52] and South Asian patients are disadvantaged if it is not identified [53] or they are unable to access services [54].

#### 3.1.5. Delivering Quality Diabetes Care and Prevention of Complications in UK South Asian Population

Individualised assessment of need and cultural sensitivity are included within the national service frameworks for diabetes, kidney disease, and care of older people [22–24] as means of delivering person centred care. The equality impact assessment for the national dementia strategy however acknowledged that although South Asians together with Black Caribbeans represent the largest ethnic minority in the UK, evidence about dementia care in these communities is lacking [55].

Research into the extent of how well healthcare services are able to meet the needs of South Asian people who have diabetes has found that whilst services have implemented the organisational element of quality improvement policy such as the Quality Outcomes Framework and shifts of diabetes care from secondary to primary care they may not have resulted in quality of care from the patient perspective [56, 57] nor in reduction of inequalities [34]. This is attributed to lack of awareness about diabetes complications and services and communication barriers in healthcare encounters and research, although studies have not specifically addressed these in connection to age and ageing.

The small number of trials testing culturally appropriate self-management programmes [58] and structured education [59] has found some short term effects on diabetes control and increased knowledge; however they conclude that more research is needed to test different types and intensities of intervention and with different South Asian groups [60]. The patient experience research referred to, however, did not specifically include older people in their inclusion criteria.

Pilots of integrated diabetes care such as the North West London Integrated Care Pilot for people over 75 years of age considered ethnicity in their design and analysis [61, 62]. The attendance by South Asian people aged 40–75 in the first year of the health checks programme was higher than previous studies of screening programmes in diverse groups highlighting the role of primary care in access for South Asian patients compared to other parts of the NHS particularly in areas with high South Asian populations with GPs who have the same ethnicity [63]. However whether the programme as a whole will achieve its target 75% uptake has been queried and the need for a combined population and high risk approach to prevention and targeting of care which considers age as the most powerful predictor of cardiovascular and diabetes risk [64] and takes into account the earlier onset of diabetes in people with South Asian ethnicity is a possible pragmatic solution [65].

#### 3.1.6. Researching the Experience of Older South Asian People with Diabetes in and across Different Settings

Patient experience research with South Asian people with diabetes in primary care identified barriers one of which was a need for information and health education to be delivered in a culturally appropriate way that matches an individual's understanding of health and disease, as well as taking into account the broader social context for ethnic minority groups and common psychological responses [66–68]. Findings related to some dimensions of ageing and South Asian ethnicity, for example, age-related expectations of health and health related behaviours, but ageing was not a specific focus of these studies although they called for multidimensional approach to understanding the preventable diabetes related mortality and morbidity.

A care pathway approach to exploring patient experience of diabetes care across primary care and specialist renal care found that South Asian patients referred to renal care lacked awareness of kidney complications of diabetes despite familiarity with diabetes over more than 10 years. Furthermore reflecting back on diabetes care patients felt there had been missed opportunities for information and self-management support [57].

The small number of studies of South Asian patients' experiences of care in secondary care kidney services also tells us more about the care of older South Asian people with diabetes as nearly half the South Asian patients requiring renal replacement therapy also have T2DM [31]. Communication difficulties are a challenge in the day-to-day provision of renal care [69] as well as for end of life care services to South Asian patients who are often older and do not speak English as their first language [70, 71].

### 3.2. Results: Part 2 Theory Building

**Figure 4:**
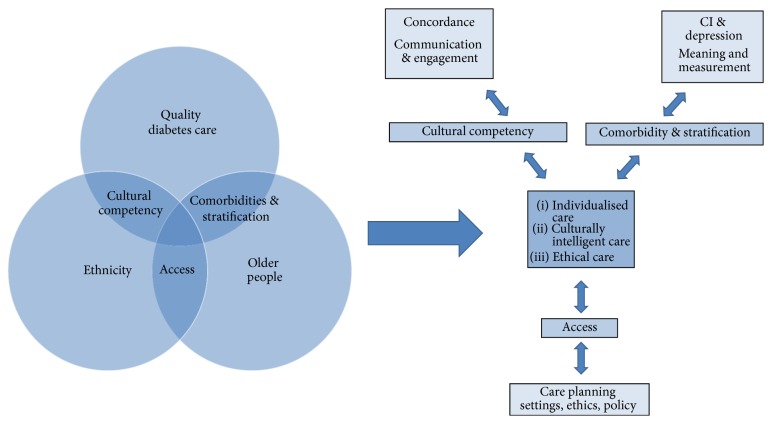
Theory building: concepts and mechanisms.

The exploratory mapping of the literature in this review created a context for the second part of our analysis. Explanatory concepts which emerged from the literature alongside the observational data were cultural stratification and comorbidities, cultural competency, and access. The relationship between these concepts and the CMO analyses in each mapped area is shown in Table 1. Together the mechanisms and explanatory concepts formed a theoretical framework (see Figure 4) for responding to the review question and identifying key areas for future enquiry which we articulated as broad research questions below.

#### 3.2.1. Comorbidities and Stratification

As diabetes complications are associated with longevity and length of time with diabetes as well as South Asian ethnicity, it is common that older South Asian people with diabetes will have multiple comorbidities requiring some sort of prioritisation and integration of treatment and care according to which conditions are of most concern or life limiting. Stratification of patients by risk, comorbidities, patient experience, and diagnosis is therefore a key part of informing effective care [72, 73] and determines the context for care.

The fact that South Asian people develop diabetes earlier and experience the complications younger means that in the context of diabetes care the descriptor “older” age needs to be brought forward relative to the White European population. The changing demographics of the UK mean that there will be more older South Asian people in the future and half will have developed diabetes by age 80 [74].

Studies which identified the cardiovascular risk and outcomes associated with diabetes and South Asian ethnicity and the small number breaking it down further into the predominant South Asian groups in the UK provide evidence for the high risk that South Asians with diabetes have for cardiovascular disease mortality and morbidity and persisting inequalities [35]. This finding is not new, but it points towards the importance of understanding the heterogeneity within ethnic categories as well as the specific genetic and social influences on health outcomes [75]. In the future it will be possible to draw more on the findings of biomarker and bariatric metabolic surgery research but at present accurate monitoring of ethnicity within the health system, the use of available data, targeting of screening programmes, and adaptability in day-to-day practice are ways of tailoring care towards individualised risk.

Detection of prediabetes, incident diabetes, and diabetes complications is important for prevention of the onset and progression of complications through the provision of appropriate and timely care which may need to be more aggressive for South Asians because of the greater risk for cardiovascular (including cognitive and renal) complications. Measures to detect complications which are culturally mediated, that is, dependent on language or ideas of dependency and quality of life, such as depression and cognitive impairment, need to be sensitive enough to identify complications in heterogeneous populations [53, 76].

The range of complications which are associated with older age and diabetes may contribute to frailty which results in vulnerability to sudden changes in health states and increased risk of falls, disability, long term care, and death [77]. A recognised frailty descriptor for the clustering of comorbidities and associated indicators has been suggested to be more meaningful in a clinical context [78, 79] than chronological age and particularly within a model for integrated care. If frailty is to be useful indicator for stratifying and tailoring diabetes care greater understanding of what it means for clinical care and prevention is required both across different ethnic groups and in relation to individual culture.

*Research Question*.* How can knowledge about diabetes comorbidities and associated impacts for older people with a South Asian background improve care that maximises quality of life and NHS resources?*

#### 3.2.2. Cultural Competency

Whereas stratification on the basis of disease, comorbidities, and symptoms dictates the context for clinical care, the way that information is conveyed and discussed to people with diabetes is important for supporting self-management and decision making in patient care.

The opportunities for prevention of diabetes and complications are an important part improving outcomes for older South Asian people with diabetes because of the earlier and extended timeframe that they are living with diabetes. The focus on primary care and integrated care as a means of delivering patient centred outcomes, if supported by systemic knowledge and awareness of culture within the NHS, aligns with the concept of culturally competent care:

*Understanding the importance of social and cultural influences on patients' health beliefs, and behaviours; considering how these factors interact at multiple levels of the health care delivery system (e.g. at the level of structural processes of care or clinical decision making); and finally, devising interventions that take these issues into account to assure quality health care delivery to diverse patient populations. [80]*

Research which investigated ethnicity and quality of diabetes care in South Asian patients in primary and secondary care identified the importance of individualising care within a culturally competent approach to support concordance in the care process [33]. For individualised care to be supported practitioners therefore not only need culturally valid tools for assessing and diagnosing comorbid conditions, but also require a culturally adaptable approach which encourages concordance, that is, mutual agreement and involvement in their care.

To achieve this one on one with patients requires the resources within the system to be in place and a full understanding of the challenge. The evidence as it stands suggests that although it is possible to target diabetes interventions [81] and make cultural adaptations these have not been shown to be cost effective or to have fully addressed motivation as a key issue which requires a better understanding of culture and healthcare interactions at an individual and family as well as organisational level. Peer support interventions have been identified as a potentially effective way of achieving culturally competent care [82] but evidence is lacking from the UK of its usefulness with particular population groups [83, 84].

The concept of cultural intelligence takes the theory of cultural competency further [85, 86] by suggesting that care providers and the healthcare system as a whole are able to work effectively with all people of any culture. On an organisational level this concerns availability of sound data to inform decisions and at the level of the clinical encounter it involves open and adaptable communication skills.

*Research Question*.* What are the most effective communication methods for promoting concordance in diabetes care with older people with a South Asian background?*

#### 3.2.3. Access

A person has access to quality care when the care they experience is meaningful and effective [87, 88]. As older people with diabetes and complications receive care in various settings: in general practice, acute departments of NHS hospitals, renal units, at home and in residential, and nursing care homes, commissioners require evidence of what constitutes quality care in these different contexts and in relation to inequalities within their local population.

Whilst the national quality improvement frameworks for diabetes and kidney services have improved diabetes care in terms of the infrastructure for monitoring in primary care with incentives for practices to do this, the evidence suggests that these do not support access to all aspects of diabetes care and that it can be fragmented and variable for all patients particularly for South Asian groups for whom there can be more barriers [89–91].

It has been estimated that a quarter of care home residents are likely to have diabetes [92] and whilst data on care home residency by ethnicity is sparse [93], it is reasonable to anticipate that numbers of South Asian older people requiring residential and social care services will grow in line with demographic changes. We also know there are growing numbers of South Asian people requiring palliative and end of life care [94] so that policy makers and commissioners must work with the range of care providers to ensure equitable access to care.

Our review of the literature highlights there is a dearth of research studies which have considered access as a collective function of providers within local systems and which include older patient and carer participation in diabetes care at local and individual levels. This is despite the growing awareness of the diabetes epidemic and observations that older age is when cultural differences and sensitivities can be most acutely experienced [95]; healthcare utilisation is at its greatest [8, 96]; and when the costs are directly felt by individuals and their carers through morbidities, disability, and reduced quality of life [97].

The prevention imperative to reduce levels of diabetes and complications requires intervention to raise public awareness of the issues of diabetes care for older people from ethnic minority groups and to shift attitudes of patients and clinicians towards a more empowered approach [98] to care planning. To enable access to holistic diabetes care for older people requires primary care commissioners to lead and facilitate an integrated approach with care providers, people with diabetes, and their carers [99].

Whilst evaluation of on-going programmes such as integrated care initiative, National Diabetes Audit with Patient Experience of Diabetes Services, and House of Care [100] will contribute to this process, primary research with patients, care providers, and formal and informal carers is necessary to understand the clinical and cultural contexts of ageing with diabetes better and to maximise ways to improve access and quality of care for older people and people with or at risk of diabetes and cardiovascular complications.

*Research Question*. What are patients and their informal and formal carers experience of involvement in care planning and how can these inform service improvement for older people living with diabetes who have a South Asian background?

## 4. Discussion

Current policy and interventions to reduce inequalities in diabetes care in older people with South Asian ethnicity have not resulted in a knowledge base of what works to reduce complications and the poorer outcomes for this population. This exploratory synthesis of the literature is the first to put forward a theory based framework for doing so.

The lack of a body of research evidence which addresses inequality and quality of diabetes care for older South Asian people with diabetes reflects many and complex relationships between diabetes and macro- and microvascular complications; the different settings where care is provided; the lack of specific inclusion of older South Asian people in research; and the heterogeneity within ethnic and age descriptors. Studies which, by default, have included this group highlighted that the ethnic specific and ageing effects of diabetes require further enquiry.

Limitations of this review relate to complexity; diffuse literature; a broad research question; and the multidimensional influence of ethnicity and culture on health. We mitigated any shortfalls in capturing relevant literature via electronic databases by hand searching and including grey literature and including broad age descriptors which was in line with the exploratory nature of this study. The realist approach taken helped to expand the knowledge base by identifying common mechanisms across different contexts which together contributed to a theoretical framework for policy, research, and practice.

It is both a strength and a limitation that our review was conducted by a team with familiarity with particular areas of the literature, that is, diabetic nephropathy and end stage renal failure in South Asians, and frailty in relation to diabetes and older people. Whilst it helped inform the search strategy and theory building it could constitute bias as published research of inequalities in diabetic kidney disease and kidney care made an important contribution and the subsequent analysis applied some of the concepts from diabetic nephropathy research previously published by two of the authors [33]. To mitigate this risk the team rereviewed the analysis and synthesis at key stages during development and invited critical analysis of the review prior to finalising the work.

Team composition comprised public health researchers and senior academics who have been involved in guideline development, some of whom are practicing clinicians, strengthened our analysis and interpretation in policy and practice terms. Theory building from such a broad question and diverse literature base identified mechanisms which were “mid-level,” conceptual, and compatible with a systems viewpoint, and interpretation into practical questions for policy makers, clinicians, and researchers was a useful element of this review (see Table 2).

Although this piece of work was limited to a UK perspective and a focus on one (albeit broad and heterogeneous) ethnic grouping, the rationale, realist methodology used, and the resulting theoretical framework could equally well be applied to other groups and other diverse populations in other countries. The focus of the review was on understanding the mechanisms which could be useful for reducing inequalities in diabetes care and because the work was exploratory the theoretical ideas are at an early stage and conceptual so also relevant to other health systems.

Figure 4 illustrates the review areas and emerging concepts and mechanisms described in the results. At the centre of this model, a theme which underpins UK diabetes policy is individualised, culturally intelligent, and ethical care for older people living and dying with diabetes. This review suggests that better understanding of how risk, disease trajectories, and comorbidities affect people differently (stratification); of how culture, and not just ethnicity, influences care (cultural competency); and of how services can be delivered so they are meaningful and effective for individuals in different settings (access) is all key mechanisms to achieve these objectives.

Our theory building went further to identify submechanisms: concordance; the use of culturally meaningful measures for comorbidities affecting older people such as depression and cognitive impairment; and care planning, in particular understanding ways that older people with diabetes can be involved to ensure that it is as person centred as possible. These submechanisms, articulated as future research questions, were at the next level of abstraction from the evidence reviewed. Addressing these will enable us to revisit and refine this early theoretical framework to further improve understanding of how to ensure equitable care at the intersection represented with a “?” in Figure 2.

Underpinning individualised care and pertinent to understanding these mechanisms is the ability of the healthcare system to work with the intersectionalities of individuals and groups within a population. The heterogeneity within broad ethnicity and age descriptors is lost in much of the research literature and a more nuanced approach to understanding individual identity and influences on health [101] will be needed to take forward the different research elements we have identified.

Research with diverse groups of older people and their care providers in different clinical and community settings requires a culturally intelligent approach by researchers [28, 102]. Conducting research with older people with diabetes also presents particular practical and ethical challenges particularly if the person has comorbidities such as cognitive impairment or is at end of life. However a focus on the lived experience and meaning of diabetes for older people with different comorbidities and cultural backgrounds is important to fill some of the evidence gaps in this area.

In practice terms too the awareness of multiple identities and individual experiences affecting diabetes care including, but not exclusive to ethnic group and age, requires closer involvement between patients and practitioners in negotiating care in order for it to be truly person centred [103]. Although this review focussed on South Asian ethnicity the mechanisms and recommendations made are transferrable and relevant to care delivery with other population groups.

In a similar way the relevance of this review in policy terms should be seen in the context of other influences on health inequalities, that is, the psychological, sociological, economic, and life course factors [104, 105]. Although we investigated inequalities and access to diabetes care in relation to ethnicity, the mechanisms identified are ways through which the diabetes care system can work with the individuals and the intersectionalities that influence diabetes risk, prevention, and management.

## 5. Conclusion

This review has found that there are very few studies which address care of older people with diabetes who have a South Asian background. As policy makers need evidence to help them respond to the changing demographic profile of the UK to commission effective services to prevent avoidable mortality and morbidity and maximise resources, this is an important limitation in the existing evidence base.

There is commissioning guidance for diabetes services and integrated care which by default covers care for chronic conditions and older people and points to earlier onset, need for services to consider ethnicity [106], but it seems that there has been limited organisational engagement, it has been low priority, and there are limited skills [107].

South Asian people experience diabetes earlier than White Europeans and have a greater risk of complications and faster progression so that care providers and patients would benefit from a better informed and targeted approach to intervention.

For policy, practice and research to make an impact on reducing inequalities in diabetes care for older people with diverse backgrounds we suggest attention is given to all three of the mid-level mechanisms: access, comorbidities, and stratification and cultural competency.

Research that specifically includes older people with a South Asian background would go some way to providing knowledge about the best way to do this.

The definition of “older” people needs to be redefined in the context of diabetes care and South Asian ethnicity and the influence of intersectionalities require more attention to understand and apply these mechanisms for reducing inequalities in diabetes care.

## Figures and Tables

**Table 1:** Proposed CMOs (context, mechanism, and outcomes) and explanatory concepts for theory building.

Mapped area of literature	Context	Mechanism	Explanatory concept cultural competency (CC), stratification (S), access (A)	Outcome
Age and South Asian ethnicity in diabetes research and policy: demography and inequality	(i) No specific focus on age and ethnicity in the research literature (ii) National frameworks and nonspecific guidelines (iii) Local level focus for interventions: community, family, primary care	(i) Cultural adaptation within services, for example, link workers (ii) Targeting cultural & social factors, for example, families, diet (iii) Ethnicity as risk factor for inequality in access	(i) CC (ii) CC, S (iii) CC, S, A	(i) Modest impacts to date, not cost effective: difficult to do & complex (ii) Cultural and social determinants can influence motivation & support for self-management (iii) Researching inequalities to include age dimension

South Asians and earlier onset of diabetes and complications	(i) Earlier onset of diabetes and complications (ii) Primary/secondary care interrelations (iii) National frameworks for quality diabetes & kidney care	(i) Quality initiatives for diabetes care in primary care (ii) Use of ethnicity data, referral patterns, progression rates (iii) System wide & pathway interventions	(i) CC, A (ii) CC, S (iii) CC, S, A	(i) Earlier diagnosis (ii) Improved diabetes care: monitoring & referral patterns (iii) Contested/better understanding quality improvements in relation to holistic diabetes care

South Asian ethnicity, heterogeneity, cardiovascular disease	(i) Heterogeneity within ethnicity (ii) Socioeconomic associations (iii) Diversity of outcomes as basis of individualised care	(i) Practice based research into stratification within diabetes populations (ii) Tailored approaches to diabetes & complications care	(i) S (ii) S, CC	(i) Targeted care (ii) Improved understanding of ethnicity influences (iii) Tackling the biological with the sociological

Diabetes and complications affecting older South Asian people	(i) Age and high prevalence of diabetes comorbidities & complications (ii) Early onset, increased risk, faster progression (iii) Extended timeframe of care	(i) System approaches: care pathway & disease trajectories (ii) Improved identification of comorbidities, for example, dementia & depression in older people with South Asian background	(i) S, A (ii) S	(i) Shift in the way we think about diabetes and ageing (ii) Prevention, identification, treatment & end of life care across different settings

Delivering quality diabetes care and prevention of complications in UK South Asian population	(i) Person centred care & assessment of need (ii) Quality care includes knowledge & information for patients (iii) Lack of evidence about cultural aspects of self-management (iv) More evidence which includes South Asian ethnicity required	(i) Cultural flexibility within care (ii) Improving access by better identification through screening (iii) Integrated care as part of a holistic and whole coordinated approach	(i) CC, A (ii) A, S (iii) S, A	(i) Better understanding of the different elements of diabetes care (ii) Access increased through combined and system wide approaches.

Researching the experience of older South Asian people with diabetes in and across different settings	(i) Lack of research involving South Asian patients (ii) Reliance on studies of ethnicity in kidney services (iii) Broader social and psychological contexts of care	(i) Culturally competent practice and research to redress inequalities in access & in research participation	(i) CC, A	(i) Improved understanding of patient experience so that care is effective and meaningful for all patients.

**Table 2:** Issues to consider in improving access to diabetes care for older people with a South Asian background.

Policy makers	Providers	Researchers
(1) There is a lack of research which has focussed on diabetes care of older people with a South Asian background. The growing numbers of older people from ethnic groups and burden of diabetes makes prevention and quality diabetes care a necessary priority for research and intervention. There is a lack participation, or access to participation, in health research studies for older people and people from ethnic minorities (including South Asian ethnicity).	Policy to be interpreted and care delivered with specific needs of older people and people from ethnic minorities in mind. Interventions need to be multilevel and system wide and promote engagement within diverse populations. NHS research and data systems to make it easier, and clinicians to be proactive to include more older people and people from ethnic minorities in research.	Researchers to develop research methodologies, methods, and skills which facilitate participation in research by older people and people from ethnic minorities.

(2) Earlier onset & progression require earlier treatment for people with South Asian background. The definition of “older” and ageing in relation to diabetes care and ethnicity can vary and this has implications for the timing of interventions.	Providers have an educative as well as treatment role so they need to be aware of differences in disease progression within diverse populations. Provider organisations and practitioners to be aware of age in relation to diabetes care with proactive in targeting timely & appropriate interventions.	Research knowledge required concerning attitudes of different providers towards prevention, older people, sociodemographics, and behaviour change. Researchers to further develop the concept of ageing in relation to diabetes care in diverse cultural groups.

(3) The complicating associations between diabetes and other chronic & preventable diseases, for example, retinopathy, depression, and dementia to be considered in policy making for older patients with South Asian ethnicity.	Providers to be aware of the impact of complications on quality of life and quality of care. Also their role in prevention through integrated and cross disciplinary services. Targeting of interventions to be based on stratification, detection, and diagnosis.	Researchers to carry out more research about complicating associations, for example, diabetes & dementia & depression. Development of culturally relevant tools (and biomarker research to pick up risk earlier).

(4) Awareness of the heterogeneity with broad ethnic groups and the requirement for adaptable and culturally intelligent services to be promoted through policy. Care planning to promote access requires an ethical and culturally intelligent approach.	Services to be flexible and communicate well with people across a cultural spectrum and also at an organisational level. Care planning in different settings, for example, end of life, care homes to involve formal and informal care providers.	Researchers to engage and communicate and engage with culturally diverse people and services. Researchers to build capacity for cross cultural and organisational health services research.
